# The Safety of Non‐immunogenic Recombinant Staphylokinase in Elderly Patients With Massive Pulmonary Embolism: A Randomized Clinical Trial FORPE

**DOI:** 10.1002/hsr2.70826

**Published:** 2025-05-19

**Authors:** Stanislav G. Leontyev, Elena B. Yarovaya, Vladimir A. Kutsenko, Oleg E. Ivlev, Anna G. Soplenkova, Andrey M. Semenov, Mikhail P. Semenov, Sergey V. Ivanov, Yulia A. Romashova, Sergey S. Markin

**Affiliations:** ^1^ N.I. Pirogov Russian National Research Medical University Moscow Russia; ^2^ National Medical Research Center for Therapy and Preventive Medicine Moscow Russia; ^3^ Lomonosov Moscow State University Moscow Russia; ^4^ Patrice Lumumba Peoples' Friendship University of Russia (RUDN) Moscow Russia; ^5^ Institute of Biomedical Chemistry Moscow Russia

**Keywords:** elderly patients, massive pulmonary embolism, non‐immunogenic staphylokinase, randomized clinical trial, thrombolytic therapy

## Abstract

**Background and Aims:**

Major bleedings are the most limiting factor of the usage of thrombolytic agents in pulmonary embolism (PE), especially in elderly patients. Non‐immunogenic staphylokinase is a recombinant staphylokinase with high thrombolytic activity, fibrin selectivity, and low immunogenic properties. We performed a post hoc analysis of safety outcomes in elderly patients with massive PE over 60 years' old who received non‐immunogenic staphylokinase in FORPE trial.

**Methods:**

A randomized, open‐label, multicenter, parallel‐group, noninferiority FORPE trial was conducted at 23 clinical sites in Russia. A total of 310 patients aged 18 years and older with massive PE proven by computed tomography pulmonary angiography, right ventricular dysfunction, and hemodynamic instability were included. The patients were randomized for treatment either the non‐immunogenic staphylokinase (15 mg) or alteplase (100 mg). Safety outcomes were hemorrhagic stroke, bleeding types 3 and 5 according to BARC classification within 7 days after randomization.

**Results:**

No cases of hemorrhagic stroke or major bleeding were registered in the non‐immunogenic staphylokinase group, whereas there were five incidences (5%) of BARC type 3 + 5 bleedings in the alteplase group (*p* = 0.03). All major bleedings and fatal hemorrhagic stroke in patients treated with alteplase were registered only in elderly patients over 60 years old.

**Conclusion:**

The FORPE trial showed that the treatment of massive PE with hemodynamic instability with the non‐immunogenic staphylokinase was safe in elderly patients over 60 years and can be used in emergency medicine in the real‐world clinical practice. Future trials and PE registries are needed to make a final decision on safety of thrombolytic therapy with the non‐immunogenic staphylokinase in elderly patients.

**Trial Registration:** ClinicalTrials.gov (NCT04688320).

## Summary

- Pulmonary embolism (PE) is globally one of the leading potentially fatal disease. For patients with massive PE and hemodynamic instability the thrombolysis can be life‐saving.
- Major bleedings are the most limiting factor of the usage of thrombolytic agents in PE, especially in elderly patients.
- New safe and effective thrombolytics are needed for treatment of massive PE. A post hoc analysis of safety outcomes of non‐immunogenic staphylokinase in elderly patients with massive PE and hemodynamic instability in FORPE clinical trial was performed.

## 1 Introduction

The most limiting factor of the usage of thrombolytic agents for the treatment of acute pulmonary embolism (PE) in elderly patients is the more than threefold increase of major bleeding risk compared with heparin‐only therapy [1]. During thrombolysis, major bleedings occurred in 5.6% in nonelderly patients, while in elderly group this rate was 2.5 times higher [2]. So, the safety of thrombolytic therapy is an urgent task of its usage in elderly patients.

Staphylokinase is a medicine for thrombolytic therapy with high biological activity [3]. Staphylokinase has a unique fibrin selectivity that made it the first‐line molecule for patients with ST‐segment elevation myocardial infarction (STEMI) and acute ischemic stroke in the 1990s. However, high immunogenic properties of staphylokinase prevented its usage. In 2012, recombinant non‐immunogenic staphylokinase with amino acid substitutions (Lys74Ala, Glu75Ala, and Arg77Ala) was created. It was shown that titers of neutralizing antistaphylokinase antibodies after the recombinant non‐immunogenic staphylokinase administration in patients with STEMI are 200 times less than after native staphylokinase usage [4].

Recently, it was shown that non‐immunogenic staphylokinase was non‐inferior to alteplase for patients with massive PE and hemodynamic instability in FORPE trial [5]. According to the primary efficacy endpoint—death from all causes within 7 days—the non‐immunogenic staphylokinase is comparable with alteplase (*p* > 0.99). In the non‐immunogenic staphylokinase group, there were no hemorrhagic stroke and major bleedings as compared with 3% of patients who received alteplase (*p* = 0.12).

Since the question of safety of thrombolytic therapy in elderly patients is actual, a post hoc analysis of major bleedings in patients who received the non‐immunogenic staphylokinase in FORPE clinical trial in patients with massive PE was performed. So, the aim of the study was to assess the safety of the non‐immunogenic staphylokinase in elderly patients with massive PE over 60 years old.

## 2 Methods

A randomized, open‐label, multicenter, parallel‐group, noninferiority trial FORPE was performed (NCT04688320). This trial included patients aged 18 years or older with massive PE according to computed tomography pulmonary angiography (CTPA) direct visualization of nonocclusive endoluminal thrombus (central filling defect completely or partially outlined by contrast agent) or of complete occlusion by thrombus in normal‐sized or enlarged vessels of one or more segmental arteries [6] with hemodynamic instability defined according to one of ESC Guidelines criteria [7] (systolic BP < 90 mmHg or systolic BP drop ≥ 40 mmHg, lasting longer than 15 min and not caused by new‐onset arrhythmia, hypovolaemia, or sepsis; systolic BP < 90 mmHg or vasopressors required to achieve a BP ≥ 90 mmHg despite adequate filling status and end‐organ hypoperfusion, or need for cardiopulmonary resuscitation). Right ventricular dysfunction measured by CTPA defined as an RV/LV ratio > 1.0 [7].

Before enrollment, all participants, their legal representatives, or medical consilium provided written informed consent. The study was conducted in accordance with all local regulations, Good Clinical Practice guidelines (as outlined at the International Conference on Harmonization), and the Declaration of Helsinki. The trial protocol was approved by the Russian Ministry of Health, No. 16 on January 15, 2019, the Ethics Committee of the Russian Ministry of Health, No. 182 on December 04, 2018, as well as local ethics committees of the clinical sites.

Non‐immunogenic staphylokinase (Fortelyzin, SuperGene LLC, Moscow, Russia) (15 mg reconstituted in 15 mL of NaCl solution [0.9%]) was administered as a single fixed intravenous bolus for all patients for 10–15 s, regardless of bodyweight. Alteplase (Actilyse, Boehringer Ingelheim, Ingelheim, Germany) was administered according to the instructions for use as an intravenous bolus of 10 mg for 1–2 min and 90 mg by continuous intravenous infusion within 2 h, maximum 100 mg. In patients weighing < 65 kg, the total dose of alteplase does not exceed 1.5 mg/kg. As recommended by current guidelines, all patients received therapeutic anticoagulation for at least 6 months [7].

The detailed description of FORPE trial design, randomization procedures, and outcomes was published previously [8].

Safety outcomes were hemorrhagic stroke, bleeding types 3 and 5 according to Bleeding Academic Research Consortium (BARC) [9] within 7 days, as well as all‐cause mortality within 30 days after randomization. All safety outcomes were assessed independently by medical staff at the clinical sites who were blinded to the trial treatment. The Safety Data Monitoring Board assessed the documentation of safety outcomes only, independently from the clinical site staff and investigators and did not collect them. All members of the Safety Data Monitoring Board vouched for the integrity and completeness of the data, and the compliance of this manuscript to the trial protocol.

### 2.1 Statistical Analysis

Statistical analysis was carried out using R (version 4.2). Continuous variables are presented as mean and standard deviation (SD) or median and interquartile range (ME [Q1–Q3]). Categorical variables are presented as *n* (%) and corresponding 95% CIs. To compare continuous variables in the dependent groups, the Mann–Whitney test was used, and to compare categorical variables, Fisher's exact test was used. For comparison of continuous variables in the dependent groups, the Wilcoxon test was used, for categorical variables—the McNemar test was used. In all used tests, we considered the two‐sided critical region. The differences were considered statistically significant if the *p* was < 0.05.

### 2.2 Role of the Funding Source

The funder of the study is the Ministry of Science and Higher Education of the Russian Federation. SuperGene LLC supplied the study drugs (both non‐immunogenic staphylokinase and alteplase). The funder had no role in data collection, data analysis, data interpretation or report writing.

## 3 Results and Discussion

**Figure 1:**
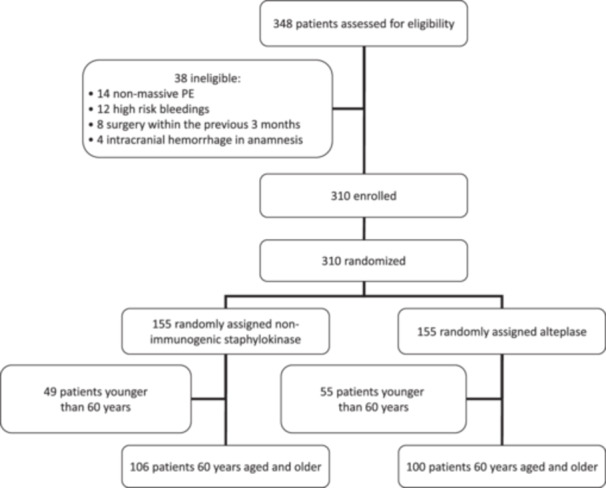
CONSORT trial profile.

Baseline demographic data, clinical characteristics, and PE risk factors were comparable between treatment arms (Table 1). In total, 106 (68%) patients in the non‐immunogenic staphylokinase group and 100 (65%) in the alteplase group were 60 years aged and older (Figure 1). The median age in the non‐immunogenic staphylokinase elderly group was 70 (66–77) years, in the alteplase elderly group—68 (64–73) years (*p* = 0.01). There was also a significant difference in the number of patients older than 75 years in the non‐immunogenic staphylokinase group compared with the alteplase group (*p* = 0.03), that had a significant impact on PESI score value and increased mortality rate.

**Table 1:** Baseline demographic data, clinical characteristics, and comorbidities.

	Non‐immunogenic staphylokinase, all group (*n* = 155)	Alteplase all group (*n* = 155)	*р*	Non‐immunogenic staphylokinase, aged 60+ (*n* = 106)	Alteplase, aged 60+ (*n* = 100)	*р*
Sex, m/f						
Male	71 (46%)	81 (52%)	0.31	43 (41%)	41 (41%)	> 0.99
Female	84 (54%)	74 (48)	0.31	63 (59%)	59 (59%)	> 0.99
Ages, years	66 (55–75)	63 (55–71)	0.07	70 (66–77)	68 (64–73)	0.01
75 years old	39 (25%)	23 (15%)	0.02	39 (37%)	23 (23%)	0.03
Bodyweight, kg	92 (19)	92 (19)	0.82	89 (18)	87 (15)	0.46
Body mass index, kg/m^2^	31.9 (6.4)	31.2 (6.1)	0.32	31.5 (6.5)	30.7 (5.6)	0.43
PE risk factors						
Deep vein thrombosis	112 (72%)	116 (75%)	0.70	82 (77%)	78 (78%)	> 0.99
Hypertension	119 (77%)	122 (79%)	0.79	95 (90%)	91 (91%)	0.82
Ischemic heart disease	27 (17%)	27 (17%)	> 0.99	25 (24%)	23 (23%)	> 0.99
Arrhythmia	21 (14%)	21 (14%)	> 0.99	18 (17%)	16 (16%)	> 0.99
COVID‐19	39 (25%)	31 (20%)	0.34	28 (27%)	17 (17%)	0.13
Diabetes mellitus	28 (18%)	29 (19%)	> 0.99	24 (23%)	24 (24%)	0.87
Dyslipidemia	25 (16%)	25 (16%)	0.12	22 (21%)	28 (28%)	0.26
Current smoker	25 (16%)	37 (24%)	> 0.99	11 (10%)	11 (11%)	> 0.99
COPD, asthma	17 (11%)	16 (10%)	> 0.99	13 (12%)	17 (17%)	0.66
Cardiovascular history						
Recurrent PE	15 (10%)	13 (9%)	0.84	13 (12%)	8 (8%)	0.36
Previous stroke	9 (6%)	10 (7%)	> 0.99	8 (8%)	7 (7%)	> 0.99
Previous myocardial infarction	4 (3%)	5 (3%)	> 0.99	4 (4%)	5 (5%)	0.74
Previous transient ischemic attack	1 (1%)	2 (1%)	> 0.99	1 (1%)	1 (1%)	> 0.99
Hip or knee replacement	4 (3%)	5 (3%)	> 0.99	4 (4%)	2 (2%)	0.68
PE characteristics						
PESI score	135 (116–150)	128 (113–145)	0.10	140 (127–154)	132 (122–153)	0.18
PESI Class I	0 (0%)	0 (0%)	> 0.99	0 (0%)	0 (0%)	> 0.99
PESI Class II	3 (2%)	5 (3%)	0.72	1 (1%)	0 (0%)	> 0.99
PESI Class III	16 (10%)	18 (12%)	0.86	7 (7%)	9 (9%)	0.61
PESI Class IV	33 (21%)	50 (32%)	0.04	16 (15%)	28 (28%)	0.03
PESI Class V	103 (67%)	82 (53%)	0.02	82 (77%)	63 (63%)	0.03
Onset to treatment time, h	27 (12–81)	17 (9–69)	0.02	31 (12–79)	16 (8–69)	0.08
Randomization to treatment time, h	15 (5–30)	15.0 (6–30)	> 0.99	15 (8–30)	15 (7–30)	0.97
Qanadli index, %	65.8 (19.8)	67.8 (19.7)	0.35	66.7 (15.8)	68.5 (17.5)	0.39
RV EDD, mm	50 (45–56)	50 (47–55)	0.95	49 (45–56)	49 (46–54)	0.71
RV/LV EDD	1.39 (1.30–1.52)	1.40 (1.34–1.51)	0.62	1.39 (1.32–1.53)	1.39 (1.33–1.52)	0.99
PASP, mmHg	60.4 (15.4)	59.9 (15.7)	0.96	62.7 (15.8)	60.8 (15.2)	0.55
Tricuspid regurgitation jet velocity, m/s	2.9 (0.9)	2.9 (0.9)	0.70	3.0 (7.7)	2.9 (0.9)	0.80
Hypokinesis of RV‐free wall (McConnell sign)	28 (18%)	24 (16%)	0.65	16 (15%)	11 (11%)	0.42
Baseline systolic blood pressure, mmHg	90 (85–100)	90 (85–100)	0.98	90 (85–100)	89 (85–90)	0.37
Baseline diastolic blood pressure, mmHg	60 (52–65)	60 (53–69)	0.47	60 (52–65)	60 (50–65)	0.97
Baseline heart rate, beats/min	110 (90–115)	110 (92–115)	0.41	110 (90–113)	109 (90–114)	0.88
Baseline respiratory rate, breaths/min	24 (20–27)	24 (21–26)	0.98	24 (20–27)	24 (22–26)	0.34
Baseline SpO_2_, %	89 (88–90)	89 (88–92)	0.30	89 (86–90)	89 (88–92)	0.04

*Note:* Data are *n* (%), mean (SD), or median (IQR).

Abbreviations: COPD = chronic obstructive pulmonary disease, EDD = end diastolic diameter, LV = left ventricle, PASP = pulmonary artery systolic pressure, PE = pulmonary embolism, PESI = pulmonary embolism severity index, RV = right ventricle.

The severity of PE risk factors was higher in elderly patients in both the non‐immunogenic staphylokinase and alteplase groups compared to all included patients. Hypertension occurred in 90% and 91% of elderly patients in the non‐immunogenic staphylokinase and alteplase group, respectively (*p* = 0.82) compared with 77% and 79% of all age patients' groups, respectively (*p* = 0.79). Both groups had a high number of deep vein thrombosis (77% and 78%, respectively, *p* > 0.99). The most of elderly patients with PE were comorbided with coronary and cerebrovascular diseases, diabetes mellitus as well as chronic obstructive pulmonary disease (COPD). All patients in both treatment arms received anticoagulation therapy for at least 6 months. Additionally, all patients received concomitant therapy (beta‐blockers, ACE inhibitors, angiotensin II receptor blockers, diuretics, antihyperlipidemic, antidiabetic agents).

Elderly patients in the non‐immunogenic staphylokinase group had a higher PESI score (140 vs. 132, *p* = 0.18) than in alteplase group. The number of patients PESI Class V with a very high risk of mortality was prevailed in the non‐immunogenic staphylokinase group, than in the alteplase group (77% vs. 63%, *p* = 0.03).

The number of COVID‐19 incidence was higher in the non‐immunogenic staphylokinase group than in the alteplase group (27% vs. 17%, *p* = 0.13).

Both the non‐immunogenic staphylokinase and alteplase elderly groups had a similar severity of PE risk factors and PE characteristics, such as massive pulmonary arteria occlusion (Qanadli index 66.7% and 68.5%, respectively, *p* = 0.39), high pulmonary hypertension (PASP 62.7 mmHg and 60.8 mmHg, respectively, *p* = 0.55) and a right ventricle dysfunction (RV/LV EDD 1.39 and 1.39, respectively, *p* = 0.99). All enrolled patients had unstable hemodynamics, tachycardia, tachypnoea, and hypoxemia (Table 1). It should be noted that the baseline systolic blood pressure was 90 (85–100) mmHg in the non‐immunogenic staphylokinase group and 89 (85–90) mmHg in the alteplase group, indicating that not all patients had a systolic blood pressure below 90 mmHg upon hospitalization. However, all patients demonstrated at least one clinical manifestation that allowed to classify their condition as hemodynamically unstable [10]. In total, 15 (14%) patients treated with the non‐immunogenic staphylokinase and 14 (14%) patients treated with alteplase had a systolic blood pressure drop more than 40 mmHg from their normal range, longer than 15 min and were not caused by new‐onset arrhythmia, hypovolaemia, or sepsis. Eleven (10%) patients in the non‐immunogenic staphylokinase group and six (6%) patients in the alteplase group required vasopressors to achieve a blood pressure more than 90 mmHg. Finally, one patient (1%) in the non‐immunogenic staphylokinase group and one patient (1%) in the alteplase group needed the cardiopulmonary resuscitation. Taken together, we may conclude that all patients enrolled in the study had a massive PE with hemodynamic instability.

The onset to treatment time was higher in the non‐immunogenic staphylokinase group compared with the alteplase group (*p* = 0.076), however, randomization to treatment time was similar in both groups (*p* = 0.97).

All‐cause mortality on Day 30 after thrombolysis was higher in the non‐immunogenic staphylokinase group in comparison with the alteplase group due to higher number of patients over 75 years of age and COVID‐19 incidences. It is known that PESI considers age as the main factor predicted mortality risk [11]. In the non‐immunogenic staphylokinase group, the number of patients with PESI Class V was significantly higher than in the alteplase group.

In a recent study, it was shown that COVID‐19 had a negative impact on blood hypercoagulation and aggravated PE course. COVID‐19 was associated with a higher incidence of PE, and in‐hospital mortality due to PE and deep vein thrombosis were correlated significantly with COVID‐19 [12]. In our study, the number of COVID‐19 incidence in the nonimmunogenic staphylokinase group was higher as compared to alteplase, that may impact on the increased mortality rate.

No cases of hemorrhagic stroke and BARC type 3 bleeding were recorded in the non‐immunogenic staphylokinase group, while there were four cases (4%) of BARC type 3 bleeding in the alteplase group (*p* = 0.05, Table 2). All these cases occurred in patients over 60 years old.

**Table 2:** Safety outcomes.

	Non‐immunogenic staphylokinase, all group (*n* = 155)	Alteplase all group (*n* = 155)	*р*	Non‐immunogenic staphylokinase, aged 60+ (*n* = 106)	Alteplase, aged 60+ (*n* = 100)	*p*
All‐causes mortality on Day 30	9 (6%)	4 (3%)	0.26	8 (8%)	4 (4%)	0.38
Major bleeding (BARC type 3 + 5) within 7 days after thrombolysis	0 (0%)	5 (3%)	0.06	0 (0%)	5 (5%)	0.03
BARC type 3 bleeding	0 (0%)	4 (3%)	0.12	0 (0%)	4 (4%)	0.05
Hemorrhagic stroke	0 (0%)	2 (1%)	0.25	0 (0%)	2 (2%)	0.23
Hematoma of the thigh	0 (0%)	1 (1%)	> 0.99	0 (0%)	1 (%)	0.48
Pulmonary hemorrhage	0 (0%)	1 (1%)	> 0.99	0 (0%)	1 (%)	0.48
BARC type 5 bleeding	0 (0%)	1 (1%)	> 0.99	0 (0%)	1 (%)	0.48
Hemorrhagic stroke	0 (0%)	1 (1%)	> 0.99	0 (0%)	1 (%)	0.48

*Note:* Data are *n* (%).

Abbreviation: BARC = Bleeding Academic Research Consortium.

There were no BARC type 5 bleedings in the non‐immunogenic staphylokinase group. One hemorrhagic stroke in 68 years old patient in the alteplase group resulted in death and was recognized as a BARC type 5 bleeding (*p* = 0.48). Thus, all major bleedings and fatal ICH in the alteplase group were registered only in patients over 60 years old.

As it was shown previously, during alteplase administration, major bleedings occurred in 7.4% of elderly patients with massive PE over 65 years old [10].

In the PEITHO clinical trial of tenecteplase in patients with intermediate‐risk PE, the major bleeding occurred in 11.5% of patients, extracranial bleeding registered in 6.3% of patients. Among all tenecteplase group, older patients had a higher rate of major extracranial bleeding than did younger patients (11.1% vs. 4.1%, respectively) [8].

According to Cochrane Database of Systematic Reviews, major hemorrhagic events occurred in 91 of 951 (9.5%) patients in thrombolysis group [13]. It is emphasized that “trials, investigating advanced reperfusion regimens and modalities for acute PE should primarily focus on early efficacy and, particularly, safety outcomes” [14]. Based on this, in the PEITHO‐3 clinical trial in patients with intermediate‐high‐risk PE a reduced dose of alteplase (0.6 mg/kg by infusion depending on bodyweight, maximum 50 mg) will be used [15]. It is expected that a reduction in the dose of alteplase will help to reduce the development of major bleeding and hemorrhagic stroke.

In the FORPE study in the non‐immunogenic staphylokinase group, there were no hemorrhagic stroke and major bleedings. Thus, we may conclude that the non‐immunogenic staphylokinase does not require dose reduction in order to prevent the incidence of major bleeding.

Non‐immunogenic staphylokinase was registered in Russia in 2012 as a medicine for STEMI treatment [16] and in 2020 for acute ischemic stroke treatment [17]. Finally, in 2023, it was registered as a thrombolytic agent for patients with massive PE.

The rapid (10–15 s) single bolus administration of non‐immunogenic staphylokinase in a fixed dose of 15 mg to all patients regardless bodyweight compared with a long‐time bolus‐infusion alteplase administration depending on bodyweight can be used in emergency medicine and can modify thrombolytic therapy in elderly patients with massive PE in the real‐world clinical practice. We speculate that the higher safety profile of the non‐immunogenic staphylokinase, the convenience of administration, a reduction in treatment delays, and advantages in cost in comparison with alteplase may allow to generalize the non‐immunogenic staphylokinase usage to countries other than Russia.

## 4 Conclusion

We have performed the post hoc analysis of the non‐immunogenic staphylokinase usage in elderly patients with massive PE in FORPE trial. It was shown that the treatment of massive PE with hemodynamic instability with non‐immunogenic staphylokinase was safe in elderly patients over 60 years.

The main limitation of the FORPE trial is a small amount of included patients. So, our data on safety outcomes are only preliminary one and the final decision on safety of thrombolytic therapy with the non‐immunogenic staphylokinase in elderly patients will be drawn in the future clinical trials and PE registries.

## Author Contributions

**Stanislav G. Leontyev:** conceptualization, writing – review and editing. **Elena B. Yarovaya:** data curation. **Vladimir A. Kutsenko:** data curation. **Oleg E. Ivlev:** data curation. **Anna G. Soplenkova:** data curation. **Andrey M. Semenov:** project administration. **Mikhail P. Semenov:** project administration. **Sergey V. Ivanov:** project administration, writing – original draft. **Yulia A. Romashova:** project administration. **Sergey S. Markin:** conceptualization, project administration, writing – review and editing. All authors have read and approved the final version of the manuscript. All authors had full access to all the data in the study and had final responsibility for the decision to submit for publication.

## Disclosure

This work was financed by the Ministry of Science and Higher Education of the Russian Federation within the framework of Agreement No. 075‐15‐2024‐643. The sponsor had no role in the design, conduct, analysis, presentation, or supervision of this paper.

## Conflicts of Interest

S.G. Leontyev has received lectureship fees from Aspen, Bayer, Medak, Pfizer, Sanofi, Servier, SuperGene, Takeda and declare a patent issued for a method for the treatment of patients with pulmonary embolism. M.P. Semenov, A.M. Semenov, and S.S. Markin declare a patent issued for a method for the treatment of patients with pulmonary embolism. All other authors report no other conflicts of interest or disclosures.

## Transparency Statement

The lead author Stanislav G. Leontyev affirms that this manuscript is an honest, accurate, and transparent account of the study being reported; that no important aspects of the study have been omitted; and that any discrepancies from the study as planned (and, if relevant, registered) have been explained.

## Data Availability

The funder of the study is committed to the responsible sharing of data from clinical trials. Data will be provided to qualified investigator on reasonable request. Deidentified participant data will be available after the publication of the results of the completed study on request to the corresponding author. Proposals will be reviewed and approved by the funder, researchers, local regulatory authorities, and the Ethics Committee of the Russian Ministry of Health. Once the proposal has been approved, data can be transferred through a secure online platform after the signing of a data access agreement and a confidentiality agreement.
